# Effects of different short-chain fatty acids (SCFA) on gene expression of proteins involved in barrier function in IPEC-J2

**DOI:** 10.1186/s40813-022-00264-z

**Published:** 2022-05-19

**Authors:** Roberta Saleri, Paolo Borghetti, Francesca Ravanetti, Valeria Cavalli, Luca Ferrari, Elena De Angelis, Melania Andrani, Paolo Martelli

**Affiliations:** Department of Veterinary Science, University of Parma, Strada del Taglio 10, 43126 Parma, Italy

**Keywords:** SCFA, IPEC-J2, Tight junctions, Intestinal epithelial barrier function, Immune response

## Abstract

**Background:**

Gut microbial anaerobic fermentation produces short-chain fatty acids (SCFA), which are important substrates for energy metabolism and anabolic processes in mammals. SCFA can regulate the inflammatory response and increase the intestinal barrier integrity by enhancing the tight junction protein (TJp) functions, which prevent the passage of antigens through the paracellular space. The aim of this study was to evaluate the effect of in vitro supplementation with SCFA (acetate, propionate, butyrate, and lactate) at different concentrations on viability, nitric oxide (NO) release (oxidative stress parameter) in cell culture supernatants, and gene expression of TJp (occludin, zonula occludens-1, and claudin-4) and pro-inflammatory pathway-related mediators (β-defensin 1, TNF-α, and NF-κB) in intestinal porcine epithelial cell line J2 (IPEC-J2).

**Results:**

The SCFA tested showed significant effects on IPEC-J2, which proved to be dependent on the type and specific concentration of the fatty acid. Acetate stimulated cell viability and NO production in a dose-dependent manner (P < 0.05), and specifically, 5 mM acetate activated the barrier response through claudin-4, and immunity through β-defensin 1 (P < 0.05). The same effect on these parameters was shown by propionate supplementation, especially at 1 mM (P < 0.05). Contrarily, lactate and butyrate showed different effects compared to acetate and propionate, as they did not stimulate an increase of cell viability and regulated barrier integrity through zonula occludens-1 and occludin, especially at 30 mM and 0.5 mM, respectively (P < 0.05). Upon supplementation with SCFA, the increase of NO release at low levels proved not to have detrimental effects on IPEC-J2 proliferation/survival, and in the case of acetate and propionate, such levels were associated with beneficial effects. Furthermore, the results showed that SCFA supplementation induced β-defensin 1 (P < 0.05) that, in turn, may have been involved in the inhibition of TNF-α and NF-κB gene expression (P < 0.05).

**Conclusions:**

The present study demonstrates that the supplementation with specific SCFA in IPEC-J2 can significantly modulate the process of barrier protection, and that particularly acetate and propionate sustain cell viability, low oxidative stress activity and intestinal barrier function.

## Background

The risk of the onset of diseases in livestock has led to the preventive use of antibiotics. This measure, in turn, can lead to the occurrence of antimicrobial resistance, which is a critical issue for both animal and human health. For this reason, the development of novel antimicrobial strategies is of crucial importance to reduce the risk of resistance onset.

In the last decades, numerous studies have aimed at finding alternative strategies to antibiotic growth promoters in pig farms [1]. Specifically, fatty acids in vivo have been extensively studied as a potential alternative to antimicrobials, in particular against *Salmonella* Enteritidis and enteropathogenic *Escherichia coli* [2, 3]. Fatty acids are essential not only for growth and health, but also for regulating metabolism and the immune system [4], as well as for enhancing intestinal epithelial barrier function [5]. Intestinal epithelial cells (IEC) represent the first line of defence against microorganisms in terms of a physical barrier and take part in innate and adaptive immune responses. The physical barrier function is provided by the presence of tight junction proteins (TJp), allowing the absorption of nutrients from the diet and preventing the transit of molecules and pathogens through the paracellular space.

TJp play important roles, as they are firstly involved in maintaining the integrity of the intestinal barrier between two adjacent epithelial cells using transmembrane proteins such as claudins (CLDN) and occludins (OCLN). Secondly, TJp are involved in maintaining the selective permeability for intracellular ions, solutes, and cellular transporters [2]. In addition, zonula occludens-1 (ZO-1), a cytoplasmic adaptor protein, is related to cytoskeletal tethering and binding of the transmembrane proteins.

It has been demonstrated that the permeability increase of the intestinal barrier can promote disorders and diseases caused by toxins and pathogens [6].

Furthermore, the integrity of the barrier is maintained by the presence of the intestinal microbiota, which, upon an optimal balance, is able to prevent pathogens from adhering to and invading the epithelial cells [7]. The roles of the microbiota are represented by the maintenance of intestinal acid–base balance, inhibition of the growth of harmful pathogens, modulation of the host intestinal immunity, and thus by the reduction of the inflammatory responses [8]. In addition, proper interactions between the microbiota, the epithelium and the mucosal immune system determine a healthy digestive ecosystem, which is maintained by the activity of microorganisms producing a wide range of bacterial metabolites, such as short-chain fatty acids (SCFA). The most abundant SCFA are acetate, propionate, and butyrate, which are used as both energy source and signalling molecules [9]. These acids are present in the intestine as end-products derived from primary fermentation of non-digestible carbohydrates under anaerobic conditions, or from secondary fermentation of SCFA themselves by bacteroides [10].

The weakly acid nature and chain length of fatty acids seem to improve several functions of the body and can be important in some physiological processes, not only related to the digestive system but also to the regulation of energy metabolism [11].

Supplied energy to colonic epithelial cells can derive from butyrate conversion to ketone bodies or carbon dioxide [12]. Furthermore, butyrate can support intestinal barrier function through a significant induction of OCLN and ZO-1 expression [13].

SCFA exert their antibacterial function by acting on different processes, including the disruption of electron transport chain at pathogen membrane level, uncoupling of oxidative phosphorylation, cell lysis, inhibition of enzyme activity, impairment of nutrient uptake, and peroxidation and auto-oxidation [14].

In weaned pigs, SCFA improve the gut barrier function, decrease the apoptosis of epithelial cells, influence intestinal DNA and barrier protein concentrations, together with nutrient absorption, intestinal growth and development [15].

In fact, these acids stimulate the relative expression of OCLN and claudin-1 (CLDN1) in the jejunum, duodenum and ileum. [15] The increase of OCLN in the jejunum of pigs upon high-SCFA concentration infusion in distal ileal suggests that SCFA could alleviate the weaning-induced damage to intestinal structural integrity by promoting TJp expression. [12]

In this study we used the porcine small intestinal epithelial cell line IPEC-J2 to evaluate the effects of an in vitro supplementation with different concentrations of SCFA (acetate, propionate, butyrate, and lactate) on viability, nitric oxide release (oxidative stress parameter) in cell culture supernatants, and on gene expression of TJp (OCLN, ZO-1, and CLDN4) and pro-inflammatory pathway-related mediators (β-defensin 1 [BD-1], tumor necrosis factor-alpha [TNF-α], and nuclear factor-κB [NF-κB]).

The IPEC-J2 cell line is a non-transformed intestinal cell line originally derived from the jejunal epithelium, isolated from a pre-colostral piglet [16]. These cells are morphologically and functionally similar to primary intestinal epithelial cells. Furthermore, IPEC-J2 showed microvilli and tight junctions [17], and can express cytokines involved in the immune response [18]. These properties made this line a suitable model for assessing and studying the direct effects of different stimuli on intestinal cells [18].

## Results

### Cell viability

ACE 2.5 and ACE 5 supplementations induced a significant linear (y_L_ = 0.045x + 0.229) and quadratic (y_Q_ = 0.016x^2 ^− 0.053x + 0.344) increase of viability in IPEC-J2 (P < 0.05) compared to the control group. Also PROP 1 and PROP 2.5 supplementations induced a significant linear (y_L_ = − 0.015x + 0.351) and quadratic (y_Q_ = − 0.015x^2^ + 0.076x + 0.245) increase of viability (P < 0.05) compared to control.

**Fig. 1:**
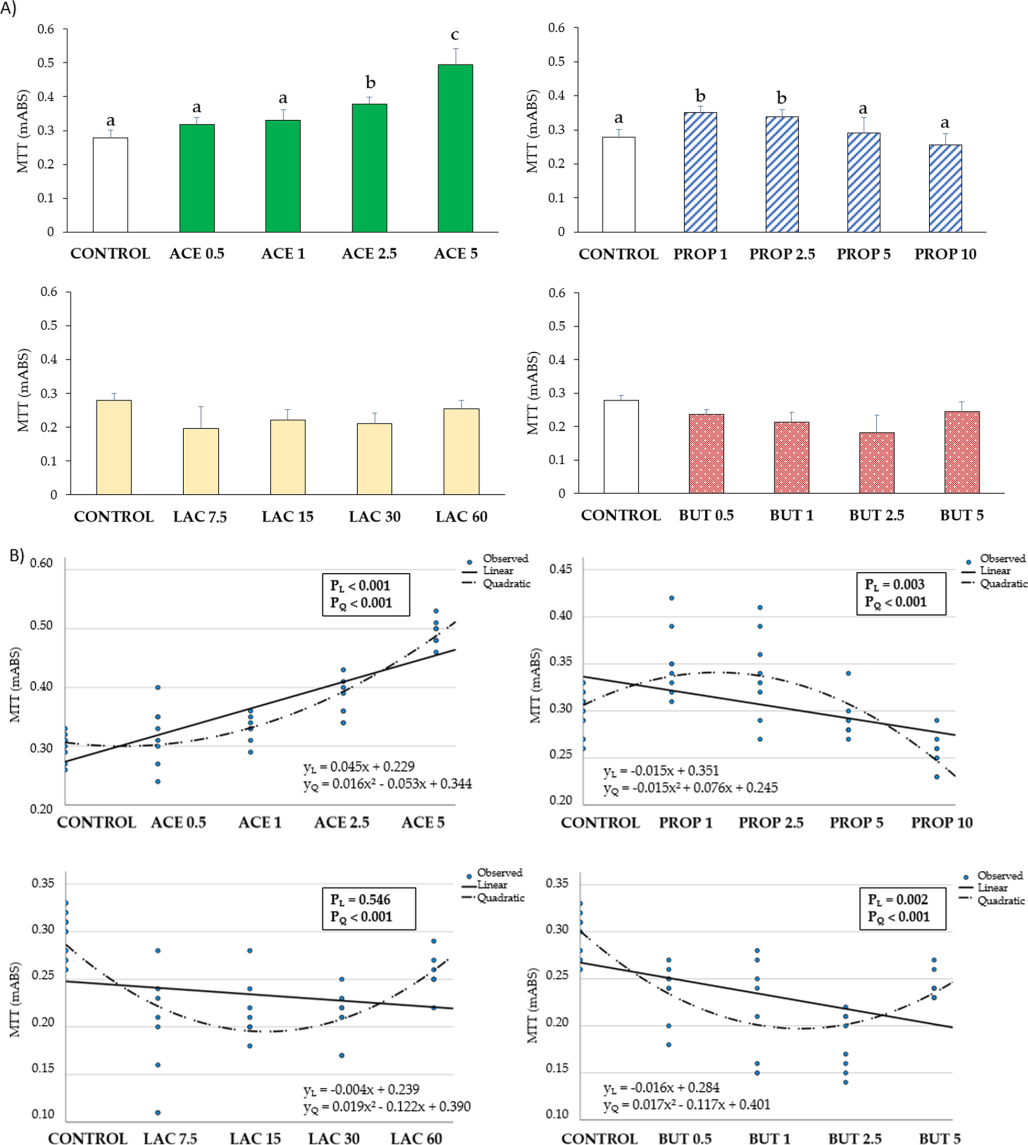
**A** Cell viability of IPEC-J2 upon medium supplementation with different concentrations of short-chain fatty acids (SCFA) at 24 h of incubation determined by an MTT assay. Each value represents the mean ± SD of 8 wells of 7 independent experiments. Different letters indicate significant (P < 0.05) differences among groups. **B** Regression graphs and analysis showing the mean values of each independent experiment and equations. y_L_: linear regression; P_L_: P-value linear regression; y_Q_: quadratic regression; P_Q_: P-value quadratic regression. The numbers in all treatment group designations refer to a mM concentration

On the other hand, lactate and butyrate showed no significant differences as compared to the control group (Fig. 1).

### Nitric oxide (NO) release

**Fig. 2:**
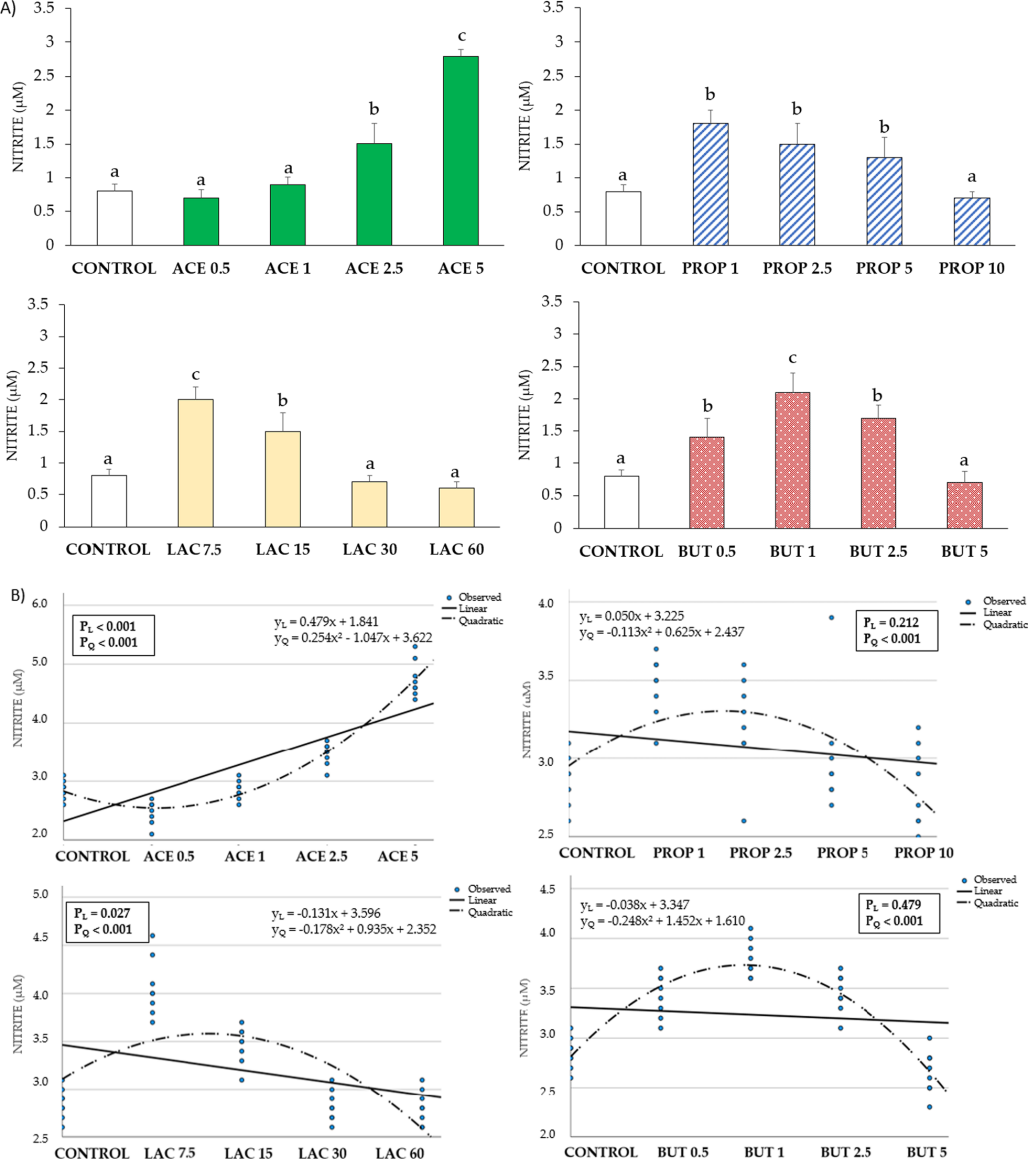
**A** Effect of the culture condition (different concentrations of SCFA) on nitrite release at 24 h of incubation. Each value represents the mean ± SD of 8 wells of 7 independent experiments. Different letters indicate significant (P < 0.05) differences among groups. **B** Regression graphs and analysis showing the mean values of each independent experiment and equations. y_L_: linear regression; P_L_: P-value linear regression; y_Q_: quadratic regression; P_Q_: P-value quadratic regression. The numbers in all treatment group designations refer to a mM concentration

Data regarding NO release are showed in Fig. 2. Acetate supplementation caused a significant linear (y_L_ = 0.479x + 1.841) and quadratic (y_Q_ = 0.254x^2 ^− 1.047x + 3.622) increase of NO (P < 0.05), with statistical differences for ACE 2.5 and ACE 5 compared to control (P < 0.05). An opposite course was showed upon lactate supplementation, which induced a linear (y_L_ = − 0.131x + 3.596) and quadratic change (y_Q_ = − 0.178x^2^ + 0.935x + 2.352) of NO release, with statistical differences for LAC 7.5 and LAC 15 compared to control (P < 0.05). Propionate induced a significant quadratic (y_Q_ = − 0.113x^2^ + 0.625x + 2.437) increase (P < 0.05), with statistical differences from PROP 1 to PROP 5 compared to control (P < 0.05). Butyrate induced a significant quadratic (y_Q_ = − 0.248x^2^ + 1.452x + 1.610) increase (P < 0.05), with statistical differences from BUT 0.5 to BUT 2.5 compared to control (P < 0.05).

### Gene expression

#### Zonula occludens-1 (ZO-1)

**Fig. 3:**
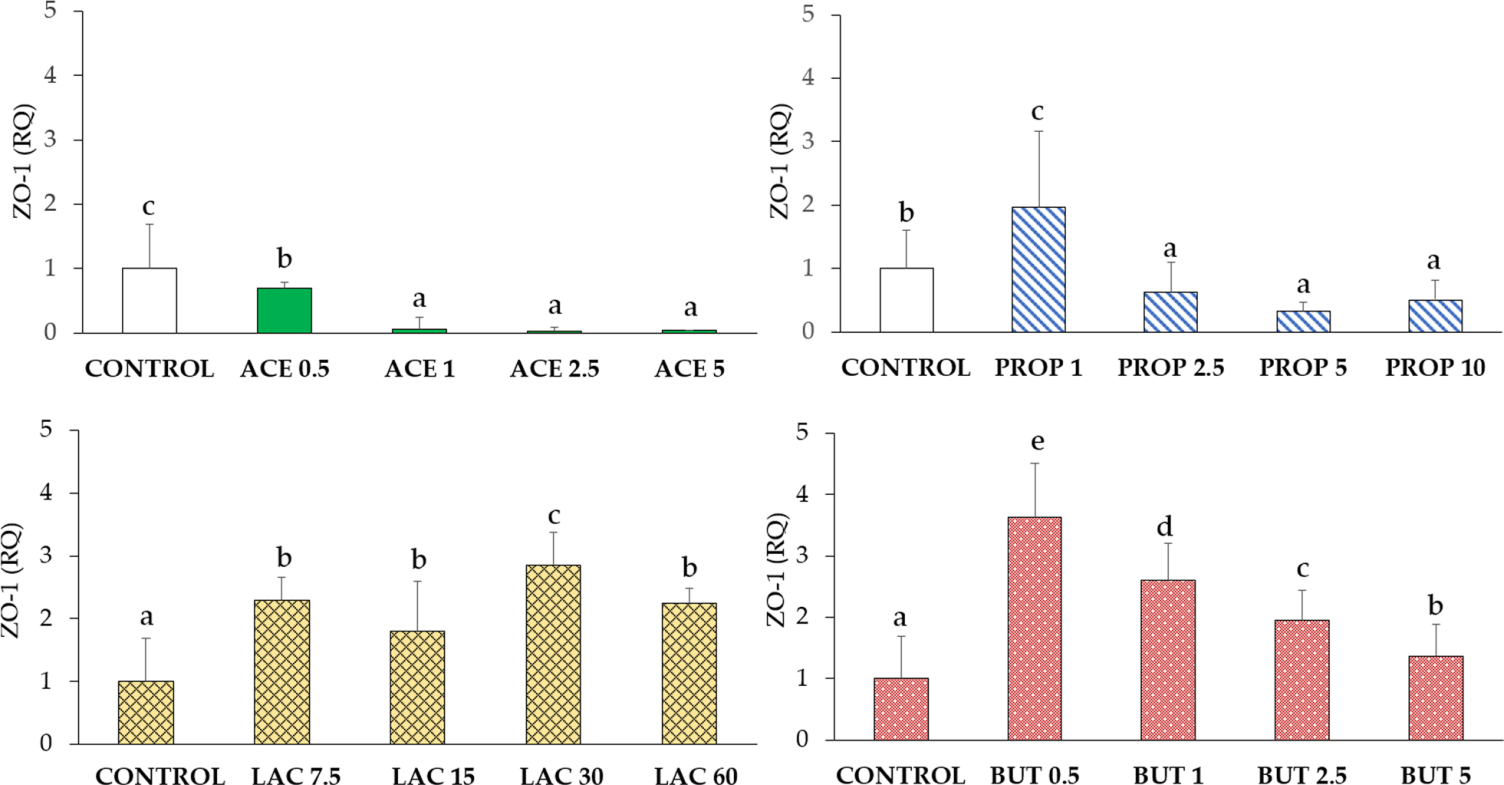
ZO-1 gene expression in IPEC-J2 cells (control) and in IPEC-J2 upon SCFA supplementation (acetate, propionate, lactate, or butyrate). Each value represents the mean ± SD of 8 replicates of 7 independent experiments. Different letters indicate significant (P < 0.05) differences among groups. Data were analyzed according to the 2^−ΔΔCt^ method in which the expression levels of the gene, normalized to the expression of the reference gene HPRT1, were expressed as relative quantities (RQ). The values were then normalized to the expression in the control group. The numbers in all treatment group designations refer to a mM concentration

A significant decrease of ZO-1 expression was detected upon supplementation with acetate in all groups (P < 0.05). In particular, a strong reduction was detected in groups ACE 1, ACE 2.5, and ACE 5 compared to control. ZO-1 expression was significantly increased in PROP 1, while higher concentrations of propionate (2.5, 5, and 10 mM) (P < 0.05) induced a strong reduction of expression as compared to the control group. Both lactate and butyrate supplementation caused a significant increase of ZO-1 expression in all groups compared to control (P < 0.05). Data are shown in Fig. 3.

#### Occludin (OCLN)

**Fig. 4:**
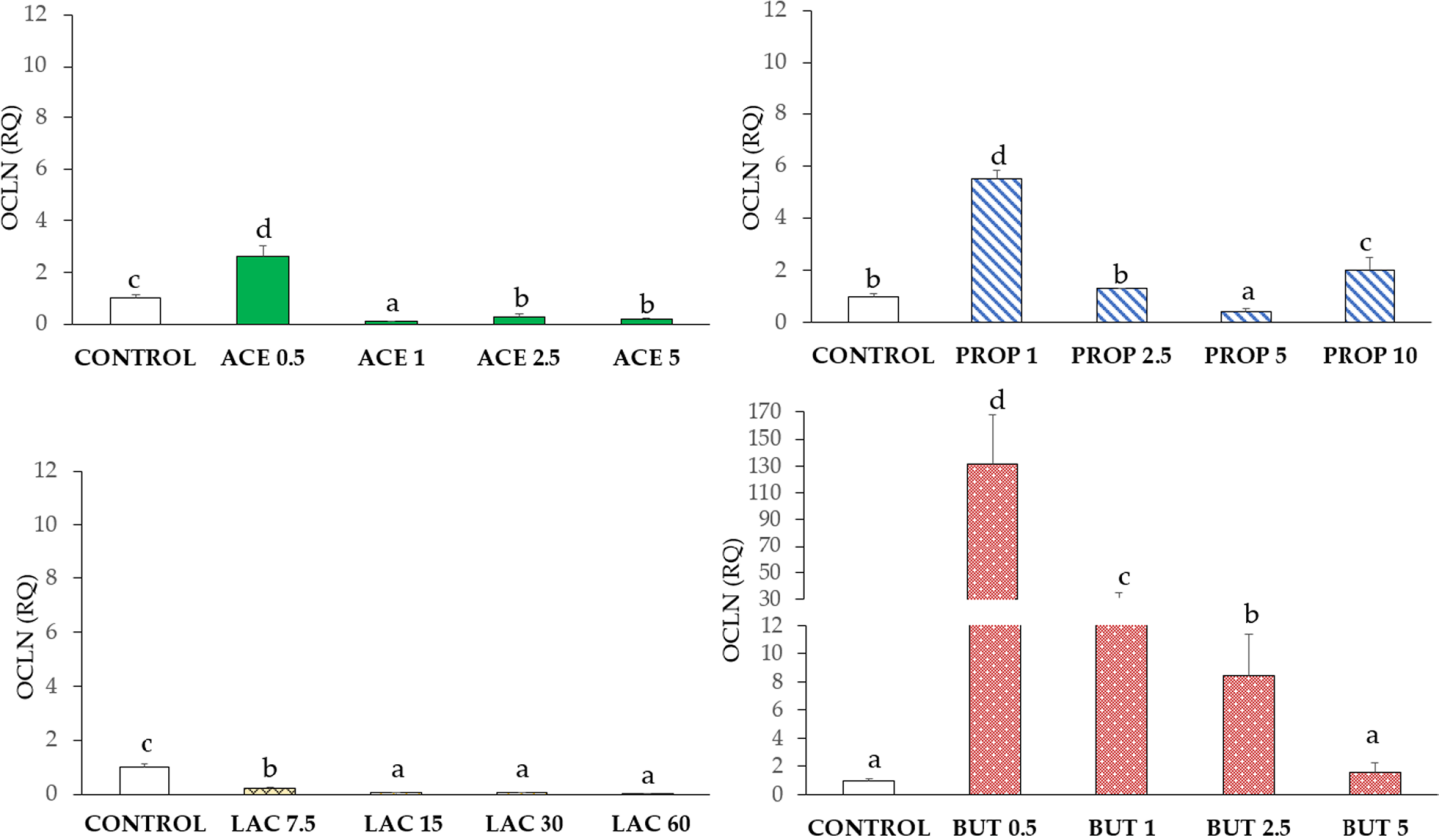
OCLN gene expression in IPEC-J2 cells (control) and in IPEC-J2 with SCFA supplementation (acetate, propionate, lactate, or butyrate). Each value represents the mean ± SD of 8 replicates of 7 independent experiments. Different letters indicate significant (P < 0.05) differences among groups. Data were analyzed according to the 2^−ΔΔCt^ method in which the expression levels of the gene, normalized to the expression of the reference gene HPRT1, were expressed as relative quantities (RQ). The values were then normalized to the expression in the control group. The numbers in all treatment group designations refer to a mM concentration

OCLN expression was significantly up-regulated in ACE 0.5 compared to control (IPEC-J2) (P < 0.05), as shown in Fig. 4. A significant decrease of OCLN expression was observed in groups ACE 1, ACE 2.5, and ACE 5 (P < 0.05) as compared to the control group. Propionate induced the highest expression of OCLN at concentrations of 1 and 10 mM (P < 0.05) as compared to control. OCLN expression was down-regulated in all groups upon lactate supplementation compared to control (P < 0.05). Upon butyrate treatment, OCLN was mostly increased at the lowest concentration tested (BUT 0.5) (P < 0.05), while no differences were detected in the BUT 5 group compared to control.

#### Claudin-4 (CLDN4)

**Fig. 5:**
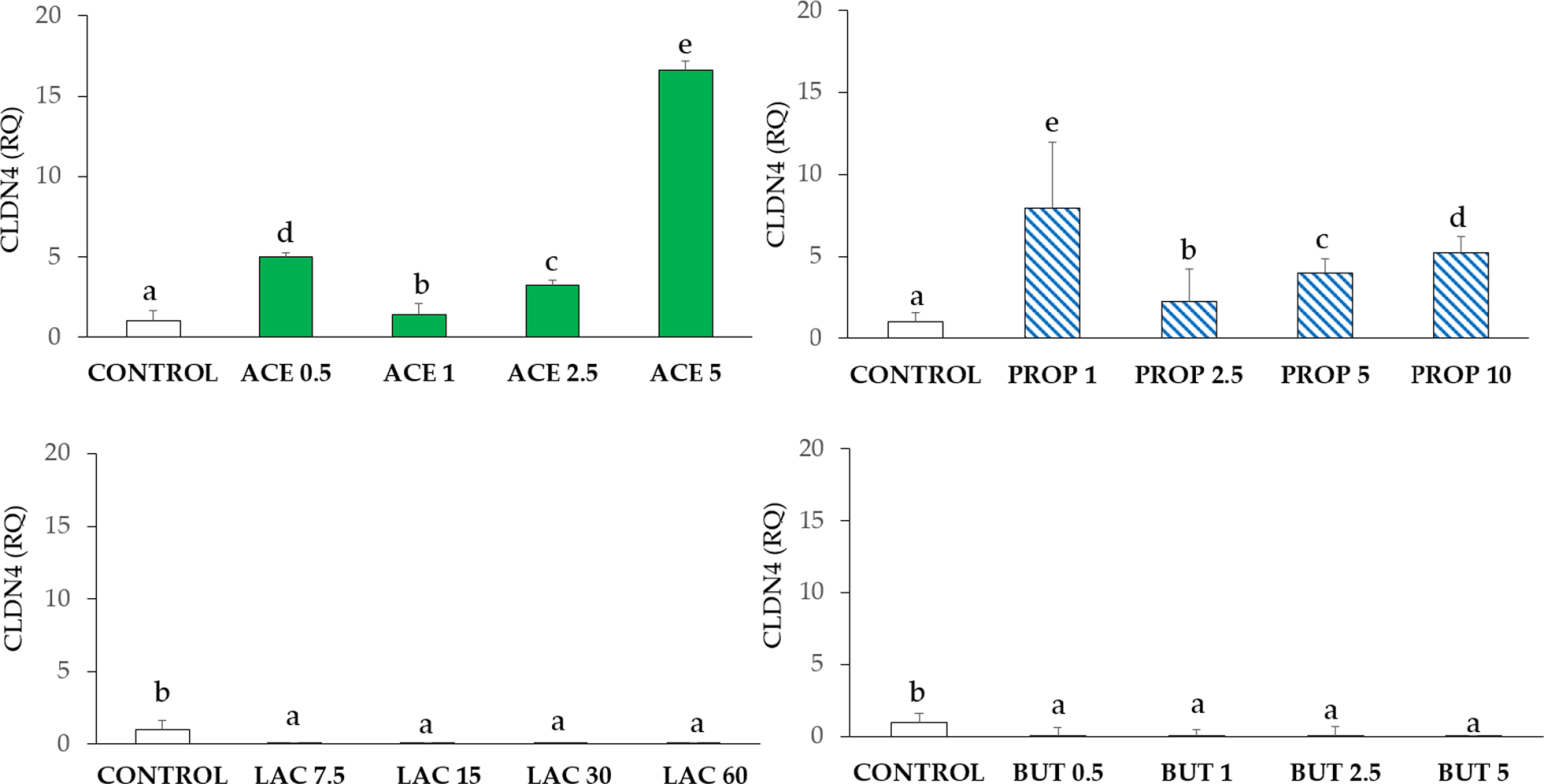
CLDN4 gene expression in IPEC-J2 cells (control) and in IPEC-J2 with SCFA supplementation (acetate, propionate, lactate, or butyrate). Each value represents the mean ± SD of 8 replicates of 7 independent experiments. Different letters indicate significant (P < 0.05) differences among groups. Data were analyzed according to the 2^−ΔΔCt^ method in which the expression levels of the gene, normalized to the expression of the reference gene HPRT1, were expressed as relative quantities (RQ). The values were then normalized to the expression in the control group. The numbers in all treatment group designations refer to a mM concentration

As shown in Fig. 5, acetate induced a significant increase of CLDN4 in all groups, in particular in ACE 5 (P < 0.05) compared to control. Also, propionate stimulated CLDN4 expression at all concentrations (P < 0.05) as compared to control. In particular, PROP 1 strongly induced CLDN4 expression (P < 0.05). Upon supplementation with both lactate and butyrate, CLDN4 expression was almost negligible, and significant differences were observed at all concentrations (P < 0.05) compared to control.

#### Nuclear factor-κB (NF-κB)

**Fig. 6:**
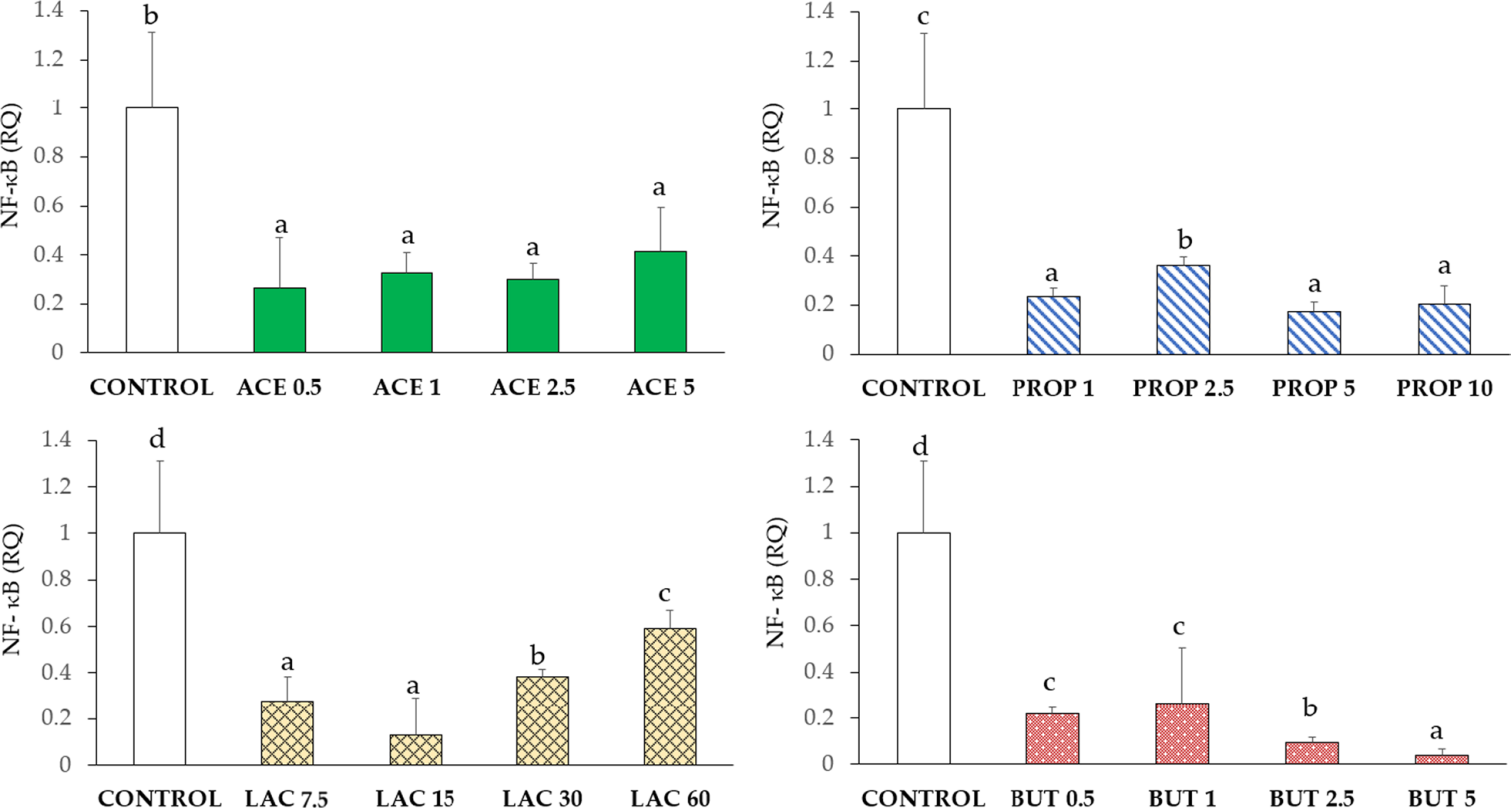
NF-κB gene expression in IPEC-J2 cells (control) and in IPEC-J2 with SCFA supplementation (acetate, propionate, lactate, or butyrate). Each value represents the mean ± SD of 8 replicates of 7 independent experiments. Different letters indicate significant (P < 0.05) differences among groups. Data were analyzed according to the 2^−ΔΔCt^ method in which the expression levels of the gene, normalized to the expression of the reference gene HPRT1, were expressed as relative quantities (RQ). The values were then normalized to the expression in the control group. The numbers in all treatment group designations refer to a mM concentration

NF-κB expression was significantly reduced (P < 0.05) upon all SCFA supplementations compared to control. Among SCFA supplementations, the highest lactate doses tested (LAC 30 and LAC 60) showed higher values of NF-κB compared to lower doses (LAC 7.5 and LAC 15) (P < 0.05). In addition, upon supplementation with butyrate, the highest NF-κB expression inhibition was observed at the highest concentration tested (BUT 5) (P < 0.05). Data are shown in Fig. 6.

#### Tumor necrosis factor-alpha (TNF-α)

**Fig. 7:**
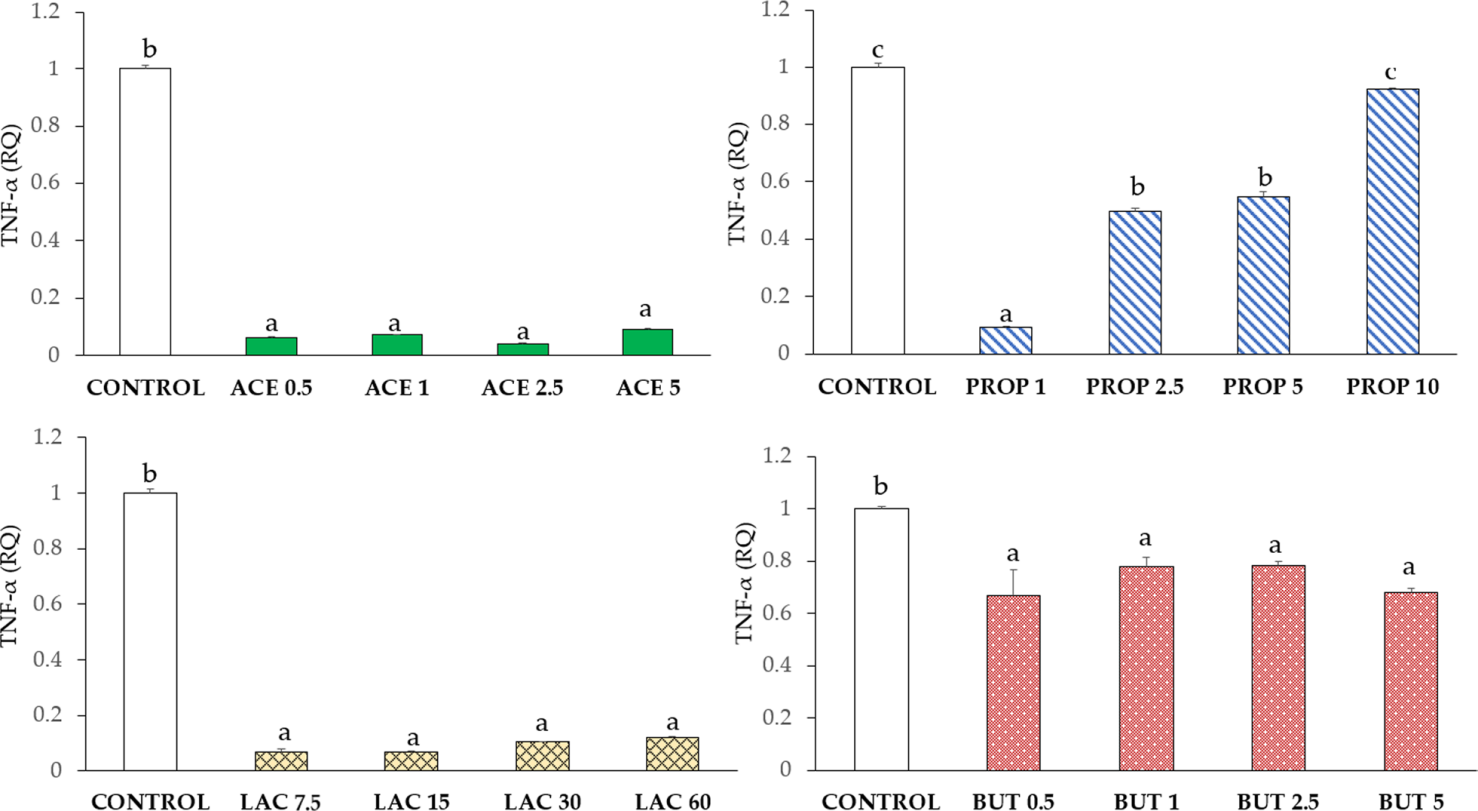
TNF-α gene expression in IPEC-J2 cells (control) and in IPEC-J2 with SCFA supplementation (acetate, propionate, lactate, or butyrate). Each value represents the mean ± SD of 8 replicates of 7 independent experiments. Different letters indicate significant (P < 0.05) differences among groups. Data were analyzed according to the 2^−ΔΔCt^ method in which the expression levels of the gene, normalized to the expression of the reference gene HPRT1, were expressed as relative quantities (RQ). The values were then normalized to the expression in the control group. The numbers in all treatment group designations refer to a mM concentration

As shown in Fig. 7, supplementation with acetate or lactate induced a very strong TNF-α down-regulation (P < 0.05) in all groups compared to control. Propionate induced a significant decrease (P < 0.05) of TNF-α expression in PROP 1, PROP 2.5, and PROP 5, as compared to control. Butyrate supplementation caused a slighter TNF-α expression decrease in all groups in comparison to control (P < 0.05).

#### β-defensin 1 (BD-1)

**Fig. 8:**
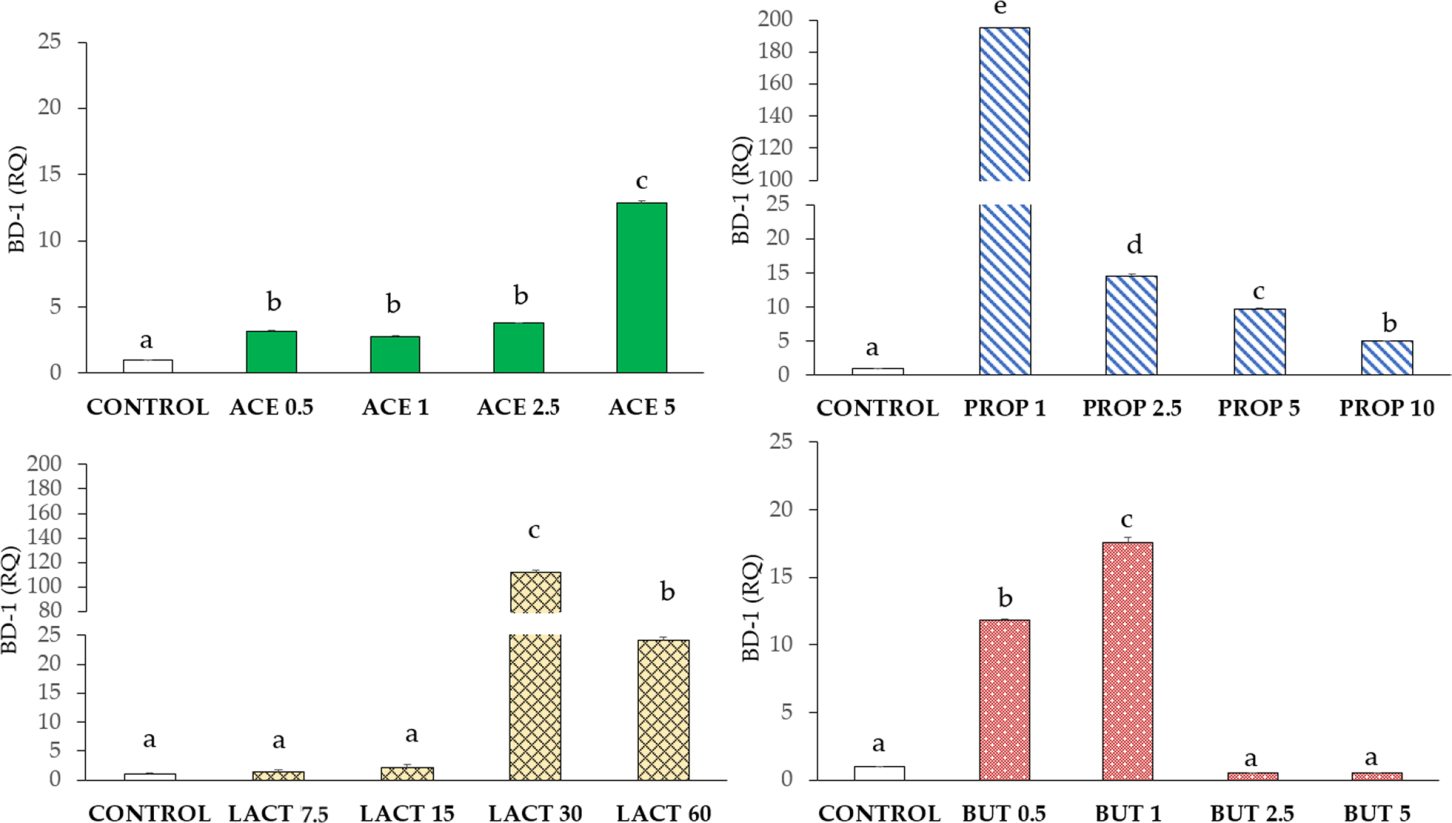
BD-1 gene expression in IPEC-J2 cells (control) and in IPEC-J2 with SCFA supplementation (acetate, propionate, lactate, or butyrate). Each value represents the mean ± SD of 8 replicates of 7 independent experiments. Different letters indicate significant (P < 0.05) differences among groups. Data were analyzed according to the 2^−ΔΔCt^ method in which the expression levels of the gene, normalized to the expression of the reference gene HPRT1, were expressed as relative quantities (RQ). The values were then normalized to the expression in the control group. The numbers in all treatment group designations refer to a mM concentration

Figure 8 shows the significant increase of BD-1 expression in all acetate and propionate groups (P < 0.05) compared to their respective controls, and opposite courses upon these supplementations. In particular, propionate 1 mM induced a 200-fold expression increase (P < 0.05). Lactate supplementation induced a strong BD-1 up-regulation in LAC 30 and LAC 60 as compared to control. Furthermore, in LAC 30, BD-1 expression was subject to a drastical 120-fold increase (P < 0.05). In addition, butyrate induced a significant increase of BD-1 expression in BUT 0.5 and BUT 1 as compared to control (P < 0.05).

## Discussion

SCFA promote the intestinal barrier function by influencing specific transcription factors and in turn, facilitating TJp assembly and mucin secretion [9, 18]. The IPEC-J2 cell line is a proper in vitro model to study the mechanisms that can occur in vivo, as this cell line is highly sensitive to nutritional stress and changes [17, 19]. The perspective is the therapeutic or dietary use of SCFA, which could decrease the intake of antibiotics, by providing energy to IEC and exerting immunomodulatory effects on intestinal inflammation, thus reducing the incidence of diarrhoea and other intestinal disorders in weaned piglets [20]. However, elevated SCFA concentrations could damage intestinal functions, therefore controversial results were obtained in vivo and in vitro on cell viability and proliferation [21, 22]. For these reasons, the present study aims to evaluate the modulating effects of different SCFA at specific concentrations in IPEC-J2, which might be useful when applied to diet, both in humans and animals. Firstly, in our study, cell viability increased upon acetate supplementation while decreased upon propionate supplementation in a dose-dependent manner. On the other hand, butyrate and lactate did not influence viability. It is known that different SCFA can exert their effects on proliferation through different mechanisms such as alteration of mitochondrial function and/or energy [23].

A change in cell viability, as highlighted by the MTT assay, was also compared to the production of NO. In fact, the same modulation of NO release and viability was observed at the corresponding concentration of acetate and propionate. We can assume that the ability of SCFA to regulate NO signalling is likely attributable to its positive effect on survival at low concentrations [24, 25]. Also, SCFA modulate NO signalling independently of their ability to inhibit histone deacetylase (HDAC) activity, like other HDAC inhibitors [26]. On the other hand, butyrate as an HDAC inhibitor, has intestinal anti-inflammatory effects [27]. This would support why increases of NO were observed in the absence of viability changes. The same effect occurred upon lactate supplementation at the lowest concentrations (7.5 and 15 mM). Therefore, upon all SCFA supplementations, a low NO release could also contribute to the beneficial effect [25].

Cell viability and the production of NO, controlled by the level of SCFA, could be related to the alteration of TJp [28]. The absence of barrier functions occurs if there is a lack of expression of claudins, which prevents the passage of luminal molecules through the paracellular pathways [6]. In our study, we chose to evaluate CLND4 expression because other claudins were not expressed in this cell line (data not shown). Increased CLND4 expression and increased cell viability were observed upon acetate and propionate supplementations. Upon lactate and butyrate supplementations, very low levels of CLDN4 were associated with unchanged viability at all concentrations. Although claudins are critical for integrity, their reduction can be compensated by occludins in long strands formation [29].

Although ZO-1 and OCLN were more expressed in the presence of lactate and/or butyrate, a greater production of NO and unchanged viability were observed. As explained above, OCLN has less influence than CLDN4 on the barrier integrity [29]. The reduced expression of ZO-1 upon acetate and propionate supplementations may be caused by the action of TNF-α, which can induce a redistribution of TJp [30]. Our data confirm the capacity of SCFA supplementation at different concentrations of improving the barrier function in intestinal epithelial cells, which in vivo react to changes in the luminal environment. In fact, SCFA play a protective effect through the maintenance of intestinal mucous barrier integrity and regulation of innate immunity [31].

Knowing that innate immunity can be strongly influenced by the presence of nutrients, the effect of SCFA was addressed by evaluating gene expression of TNF-α, NF-κB, and BD-1. NF-κB, as a transcription factor, plays an important role in regulating the expression of genes coding for cytokines in immune and inflammatory responses [32]. TNF-α is known to induce the transcription of genes regulating proliferation and differentiation, cell survival, and the inflammatory cascade [33].

The reduction of TNF-α secretion was previously observed as determined by butyrate supplementation, which induces the inhibition of NF-κB activation and the degradation of the intestinal barrier [32]. Our results demonstrate that in vitro supplementation with acetate, propionate, lactate, and butyrate can inhibit TNF-α and NF-κB expression.

Furthermore, an important mechanism of innate immune response is related to the release of antimicrobial peptides into the lumen of the gastrointestinal tract, which allows to directly kill bacterial and viral pathogens[34]. In our study we considered BD-1, a member of the antimicrobial peptides family. The pattern of BD-1 expression also reflects membrane proteins expression (ZO-1, OCLN, and CLDN4), which could confirm the role of defence in the maintenance of barrier integrity [35]. Overall, our data highlight that BD-1 expression has the same course as CLDN4 upon acetate and propionate supplementations. Moreover, BD-1 expression showed comparable responses with ZO-1 upon lactate and butyrate supplementations. Although BD-1 was proved to inhibit in vivo the pro-inflammatory cytokine cascade, involving TNF-α secretion, and the downregulation of the MAPK and NF-κB signalling pathways [34], our results do not support a correlation between BD-1, TNF-α, and NF-κB gene expression.

In summary, each SCFA tested has a different effect, and this effect is dependent on a specific concentration or concentration range. Specifically, acetate and propionate resulted to improve viability and maintenance of barrier integrity at 2.5–5 mM, and 1 mM, respectively. On the other hand, lactate and butyrate showed a predominant effect on barrier protection at 30 mM and 0.5–1 mM, respectively.

## Conclusions

Despite SCFA are widely employed in the pig diet, the use based on the different actions of each SCFA would be desirable. Our data highlight the positive in vitro effect of SCFA on intestinal viability and maintenance of intestinal integrity, which are dependent on the type and concentration of SCFA. In our opinion, their use could be considered both individually and as combination to enhance their effects.

## Materials and methods

### Cell cultures and culture conditions

Initially, IPEC-J2 were cultured in flask in Dulbecco’s Modified Eagle Medium/Ham’s F-12 (DMEM/Ham’s F-12) (Merck; Darmstadt, Germany) + 5% fetal bovine serum (FBS) (ThermoFisher; Carlsbad, CA, USA), supplemented with 5% penicillin/streptomycin/amphotericin B, glutamine (2 mM) (Merck; Darmstadt, Germany) in a humidified environment at 37 °C, 5% CO_2_. The number of IPEC-J2 cells was determined using a haemocytometer and cell viability (never less than 95%) was assessed by Trypan blue (0.1%) (Merck; Darmstadt, Germany) exclusion. After 24 h, at about 80–90% confluence, IPEC-J2 cells were trypsinized and incubated for 24 h in 24-well cell culture plates (BD Falcon, Corning Inc., Corning, NY) at a density of 1 × 10^5^ cells/well/ml in DMEM/Ham’s F-12 + 5% FBS or in the same medium supplemented with SCFA as reported below. Cells were used between passage 28 and 30.

The experimental groups were the following:

1. IPEC-J2 in DMEM/Ham’s F-12 medium (group: CONTROL);
2. IPEC-J2 in DMEM/Ham’s F-12 with 0.5, 1, 2.5, or 5 mM of sodium acetate (Merck; Darmstadt, Germany) (groups: ACE 0.5, 1, 2.5, 5);
3. IPEC-J2 in DMEM/Ham’s F-12 with 1, 2.5, 5, or 10 mM of sodium propionate (Merck; Darmstadt, Germany) (groups: PROP 1, 2.5, 5, 10);
4. IPEC-J2 in DMEM/Ham’s F-12 with 7.5, 15, 30, or 60 mM of sodium lactate (Merck; Darmstadt, Germany) (groups: LAC 7.5, 15, 30, 60);
5. IPEC-J2 in DMEM/Ham’s F-12 with 0.5, 1, 2.5, or 5 mM of sodium butyrate (Stemcell Technologies; Vancouver, Canada) (groups: BUT 0.5, 1, 2.5, 5).

The ranges of SCFA concentrations were chosen based on data reported in literature. It was chosen to narrow acetate, butyrate, and propionate ranges as no benefits were observed at high concentrations. For lactate, it was chosen to narrow the range and define an optimal concentration [19, 36, 37].

### IPEC-J2 viability assay

Cell viability was determined by a 3-(4,5-dimethylthiazol-2-yl)-2,5-diphenyltetrazolium bromide (MTT) (Merck; Darmstadt, Germany) colorimetric assay as previously described [38]. Briefly, IPEC-J2 were seeded into 96-well plates at a density of 1 × 10^4^ cells/well for 24 h in 200 μL of complete culture medium or SCFA-supplemented medium. MTT assays were performed by adding 20 μL (5 mg/mL) of MTT labelling solution to IPEC-J2 and incubating for 4 h. Afterwards, the medium was removed and IPEC-J2 were lyzed with 150 μL dimethyl sulfoxide (DMSO) (Merck; Darmstadt, Germany) in order to solubilize the purple formazan crystals for detection at 490 nm by using a Victor-3^™^ 1420 Multilabel Counter (PerkinElmer, Waltham, MA, USA).

### Nitric oxide (NO) assay

NO production was assessed by measuring the amount of nitrite (NO_2_), a stable metabolic product of NO, in the culture medium by the Griess reaction after 24 h of IPEC-J2 culture by using a Victor-3 Multilabel microplate counter (Perkin Elmer, Inc., CA, USA), as previously reported [38].

### RNA extraction and reverse transcription (RT)

Total RNA was isolated from about 1 × 10^6^ IPEC-J2/well using a TRI-reagent^™^ solution (ThermoFisher; Carlsbad, CA, USA) according to the manufacturer’s instructions and reverse-transcribed to generate complementary DNA (cDNA) using oligo-dT primers (Bioneer; Daejeon, Korea); purity (260/280 nm ratio) and concentration (at 260 nm) were assessed using a BioSpectrometer^®^ (Eppendorf AG, Hamburg, Germany). RNA samples were DNAse-treated (Merck; Darmstadt, Germany) and 1 µg/20 µL was reverse-transcribed using a HiScript III Rt SuperMix (Vazyme Biotech Co.; Nanjing, China). RT was performed using a StepOne^™^ thermocycler (Applied Biosystems, StepOne software v. 2.3) and, according to the manufacturer’s instructions, under the following thermal conditions: 2 min at 45 °C, 15 min at 37 °C, followed by 5 s at 85 °C. The cDNA samples were stored at − 20 °C.

### Real-time quantitative PCR (qPCR)

The cDNA samples were used as a template for real-time quantitative PCR (qPCR) performed by using a StepOne^™^ thermocycler (Applied Biosystems, StepOne^™^ software v. 2.3).

The cDNA (20 ng/20 µL) was amplified in duplicate using a Fast Power-Up^™^ SYBR^®^ Green Master Mix (Applied Biosystems; Foster City, CA, USA) and specific primer sets (Eurofins Genomics; Ebersberg, Germany) for ZO-1 at 400 nM and for the other genes at 300 nM. Specifics of each primer set for identification of gene expression are reported in Table 1. Samples were kept at 95 °C for 20 s (hold step) to allow DNA-polymerase activation and then subjected to 40 cycles consisting of a denaturation step at 95 °C for 3 s followed by an annealing/extension step at 60 °C for 30 s. The reference hypoxanthine phosphoribosyltransferase-1 (HPRT-1) gene [39] was selected among other tested reference genes (i.e., glyceraldehyde-3-phosphate dehydrogenase (GAPDH), β-2-microglobulin (β-2MG) [31], and 18S rRNA [40]) as endogenous control according to minimal intra-/inter-assay variation and based on previous results [41,42]. Data were analyzed according to the 2^−ΔΔCt^ method [43] in which expression levels of each gene, normalized to the reference gene HPRT-1 cDNA amount and expressed as relative quantities (RQ), were calculated with regards to the expression level in IPEC-J2 in DMEM/Ham’s F-12 medium at 24 h. A melting curve analysis for specific amplification control was performed (60–95 °C) at the end of the amplification cycles. No-RT controls and no-template controls (NTC) were included, and the latter were assumed as negative and reliable if the quantification cycle (Cq) was ≥ 35.

**Table 1:** Target genes and details of the primer sequences used for quantitative SYBR^®^ Green real-time PCR amplification. The HPRT-1 gene was used as endogenous control gene

Target gene	GenBank accession nr	Primer sequence	Efficiency (%)	Slope	r^2^	Amplicon length (bp)
BD-1 [44]	NM_213838	F 5′-CAGCCACCAGCATGAGACT-3′ R 5′-CAGGTAACAGGACCATGAGGA-3′	101.47	− 3.28	0.99	63
CLDN4 [44]	AB235916	F 5’-TATCATCCTGGCCGTGCTA-3’ R 5’-CATCATCCACGCAGTTGGT-3’	100.2	− 3.29	0.99	71
OCLN [45]	FN400888	F 5’-GGAGTGATTCGGATTCTGTCTATGCT-3’ R 5’-CGCCTGGGCTGTTGGGTTGA-3’	103.8	− 3.03	0.99	423
ZO-1 [45]	CV870309	F 5′-GGCGCACGGCGAAGGTAATT-3′ R 5′-CTATCAAACTCAGGAGGCGGCACT-3’	103.7	− 3.21	0.98	405
NF-κB [44]	DQ834921	F 5’-GAAGGACCTCTAGAAGGCAAAA-3’ R 5’-GCTTTGGTTTATGCGGTGTT-3’	104.2	− 3.22	0.99	63
TNF-α [46]	NM_214022	F 5’-ACTGCACTTCGAGGTTATCGG-3’ R 5’-GGCGACGGGCTTATCTGA-3’	98.5	− 3.36	0.99	118
HPRT-1 [44]	DQ845175	F 5’-ACACTGGCAAAACAATGCAA-3’ R 5’-TGCAACCTTGACCATCTTTG-3’	102.01	− 3.25	0.99	71

*HPRT-1* hypoxanthine phosphoribosyltransferase-1, *CLDN4* claudin-4, *OCLN* occludin, *ZO-1* zonula occludens-1, *BD-1* β-defensin 1, *NF-κB* nuclear factor-κB, *TNF-α* tumor necrosis factor-alpha, *F* forward, *R* reverse, *bp* base pairs

## Statistical analysis

Each experiment was repeated for seven times and each culture condition was performed in eight replicate wells. Data were analysed by ANOVA (IBM^®^ SPSS^®^ Statistics v.28, NY, USA) using a model with group, and interaction between groups as fixed factors. The least significant difference (LSD) post-hoc test was used to compare means when significant differences (P < 0.05) were found. Furthermore, linear regression (y = ax + b) and quadratic regression (y = ax^2^ + bx + c) were fitted by determining the linear and quadratic effects of SCFA concentrations on IPEC-J2 viability and NO release and considered significant if P < 0.05. Pearson’s correlation analysis was carried out for all markers. Experimental data were presented as means ± standard deviation. Differences among groups were considered significant if P < 0 0.05.

## Data Availability

All data will be available from the corresponding author upon reasonable request.
